# Transmission of *Cricket paralysis virus* via exosome-like vesicles during infection of *Drosophila* cells

**DOI:** 10.1038/s41598-018-35717-5

**Published:** 2018-11-26

**Authors:** Craig H. Kerr, Udit Dalwadi, Nichollas E. Scott, Calvin K. Yip, Leonard J. Foster, Eric Jan

**Affiliations:** 1 Department of Biochemistry and Molecular Biology, Life Sciences Institute, University of British Columbia, Vancouver BC, V6T 1Z3, Canada; 2 Michael Smith Laboratories, University of British Columbia, Vancouver BC, V6T 1Z3 Melbourne, Australia; 3 Department of Microbiology and Immunology, University of Melbourne, Melbourne, Australia

## Abstract

Viruses are classically characterized as being either enveloped or nonenveloped depending on the presence or absence of a lipid bi-layer surrounding their proteinaceous capsid. In recent years, many studies have challenged this view by demonstrating that some nonenveloped viruses (e.g. hepatitis A virus) can acquire an envelope during infection by hijacking host cellular pathways. In this study, we examined the role of exosome-like vesicles (ELVs) during infection of *Drosophilia melanogaster* S2 cells by Cricket paralysis virus (CrPV). Utilizing quantitative proteomics, we demonstrated that ELVs can be isolated from both mock- and CrPV-infected S2 cells that contain distinct set of proteins compared to the cellular proteome. Moreover, 40 proteins increased in abundance in ELVs derived from CrPV-infected cells compared to mock, suggesting specific factors associate with ELVs during infection. Interestingly, peptides from CrPV capsid proteins (ORF2) and viral RNA were detected in ELVs from infected cells. Finally, ELVs from CrPV-infected cells are infectious suggesting that CrPV may hijack ELVs to acquire an envelope during infection of S2 cells. This study further demonstrates the diverse strategies of nonenveloped viruses from invertebrates to vertebrates to acquire an envelope in order to evade the host response or facilitate transmission.

## Introduction

Traditionally, viruses have been categorized into one of two classes: enveloped or nonenveloped^1^. Envelopes are acquired through non-lytic release from the host cell whereby the virus typically ‘buds’ from a distinct membrane in the cell. For example, hepatitis C virus, hantavirus, and influenza virus bud from the ER, Golgi, and plasma membrane, respectively^2–4^. Envelopes provide the virus advantages including the ability for the virus to circulate and avoid immune detection. Peplomers embedded within an envelope render it indispensable for those viruses that harbor one as it provides key determinants for cell tropism and mechanism of entry into host cells. By contrast, the absence of an envelope bestows its own advantages; nonenveloped viruses are characteristically more resistant to chemical treatments and have greater environmental stability, thus allowing them to persist outside of a host for longer. Viruses belonging to this class, such as adenovirus and picornaviruses, assemble and accumulate in nonlumenal compartments until the host cell membrane is dismantled leading to release of the progeny virions. Whether a virus has evolved to possess an envelope or not has a substantial impact on how it is transmitted and recognized by the host immune system.

The classification of enveloped or nonenveloped viruses has been recently challenged by surmounting evidence demonstrating non-lytic spread of several nonenveloped viruses in both tissue culture and animal hosts^5–8^. Recent work demonstrated that poliovirus, coxsackievirus B3, and rhinovirus usurp the autophagic pathway to package virions *en bloc* into a single bilayer phosphatidylserine-containing vesicles that are non-lytically released from the host cell^6^. It is proposed that a double-membraned vesicle wraps the virions and then fuses with the plasma membrane, therefore releasing a virion with a single bilayer. This mode of transmission is thought to increase infection efficiency and may permit genetic complementation between quasi-species. In contrast to poliovirus and CVB3, hepatitis A virus (HAV), another member of the *picornaviridae*, hijacks the exosomal pathway to acquire an envelope for non-lytic release^5^. HAV interacts with ALIX to use the ESCRT-III complex to drive sorting of HAV virions into multivesicular bodies (MVBs), thus leading to secretion of membranous virions from the host cell. The envelope acquired by HAV offers the advantage of cloaking it from neutralizing antibodies that circulate in the host blood leaving the virus able to spread from cell-to-cell. Nonenveloped HAV virions are found in the stool of infected chimpanzees, suggesting that the acquisition of an envelope is not a passive mechanism and may be cell type specific^5^.

In general, exosomes are small (30–100 nm) vesicles of endocytic origin that are secreted by all mammalian cell types. They have been shown to contain active enzymes, metabolites, mRNAs, and small RNAs that are transported to adjacent cells^9,10^ and thus, exosomes are thought to participate in cell-to-cell communication. Exosomes play a significant role during viral infection by facilitating both the host immune response and viral pathogenesis^11,12^. Interestingly, exosome-like vesicles are also secreted by virus-infected insect cells, suggesting an ancestral cell-to-cell communication pathway^13–15^. A recent report demonstrated that exosome-like vesicles are secreted by haemocytes in virus-infected fruit flies that contain virally-derived siRNAs, which are then delivered to cells throughout the fly eliciting a long-lasting systemic RNAi response^16^. Still, little is known if insect viruses, much like their mammalian counterparts, can utilize extracellular vesicles to their own advantage.

Here, we investigate the role of exosome-like vesicles in *Drosophila* S2 cells during cricket paralysis virus (CrPV) infection. CrPV is a member of the *dicistroviridae* family that includes *Drosophila* C virus and Israeli acute paralysis virus. Dicistroviruses have served as a model for understanding virus host interactions in invertebrates such as viral translational controls and antiviral responses^17,18^. CrPV is a positive-strand RNA virus ~9 kb in length that encodes two main open reading frames. Translation of the CrPV ORFs is mediated by distinct internal ribosome entry sites (IRES); the 5′untranslated region (5′UTR) IRES directs the first open reading frame (ORF1) which encodes the viral non-structural proteins such as the RNA-dependent RNA polymerase (RdRp) and the 3C-like protease and the intergenic (IGR) IRES drives ORF2 which encodes the viral structural proteins^18^. The life cycle of CrPV infection in S2 cells is relatively fast; after virus adsorption, CrPV infection leads to cytopathic effects by 10–12 hours post infection (hpi). Egress of CrPV is thought to primarily to occur via lytic release; however, it has not been examined in detail whether alternative egress paths exist. In this study, we examined the proteomes of ELVs from mock- and CrPV-infected cells and reveal that CrPV structural proteins are present in ELVs. We demonstrate that CrPV, in addition to lytic release, appears to hijack exosome-like vesicles for non-lytic release to promote viral infection within *Drosophila* cells.

## Materials and Methods

### Cell culture and virus

*Drosophila* Schneider line 2 (S2) cells were maintained and passaged in Shield’s and Sang medium (Sigma) supplemented with 10% fetal bovine serum depleted in exosomes at 25 °C. Exosomes were depleted by spinning FBS at 120,000 relative centrifugal force (RCF) for 18 h at 4 °C. Propagation and purification of CrPV in *Drosophila* S2 cells has been previously described^19^.

Viral titres were determined as previously described^19^. Briefly, a total of 1.5 × 10^6^ S2 cells we incubated with serial dilutions of infected-cell supernatant for 30 min, resuspended in media, and added into a 96-well plate coated with concavilin A (0.5 mg/mL; Calbiochem). Plates were incubated at 25 °C for 8 h. Cells were then washed with PBS before fixation with 3% paraformaldehyde for 15 min followed by methanol permeabilization for 10 min. Fixed cells were incubated with an anti-ORF2 antibody (1:250 dilution in 5% bovine serum albumin in PBS) for 1 h at room temperature. Subsequently, cells were washed three times with PBS and incubated with a Texas Red IgG anti-rabbit (1:500 dilution in 5% bovine serum albumin in PBS; Invitrogen) for 1 h at room temperature. Finally, cells were washed with PBS and stained with Hoechst dye (0.5 µg/mL). Plates were analyzed with a Cellomics Arrayscan HCS instrument and the number of infected cells was used to determine viral titres. Each titre is the result of at least three replicate experiments.

### Cells infections with CrPV

For all infections involving CrPV, S2 cells were first pelleted at 200 RCF for 5 min before being resuspended in 100 uL of PBS containing a corresponding amount of virus (e.g. for MOI 10, 1.0 × 10^6^ cells were infected with 1.0 × 10^7^ FFU of CrPV). CrPV was allowed to adsorb for 30 min at 25 °C with agitation. Subsequently, cells were washed twice with 1X PBS, pelleted, resuspended in exosome-depleted media, and incubated at 25 °C.

### Exosome isolation

Exosomes were isolated from S2 cells as previously described^13^. 5.0 × 10^7^ cells were cultured in 50 mL of exosome-depleted media for 24 h. Cells and media were harvested and cells were pelleted at 300 RCF for 5 min. Cells were kept for further analysis downstream. Media was cleared of cellular debris by serial centrifugation at 5000 RCF for 10 min followed by 10,000 RCF for 30 min both at 4 °C. The supernatant was then under laid with a 5 mL 45% sucrose cushion and ultracentrifuged at 100,000 RCF for 2 h at 4 °C. The interphase containing the exosomes was kept by removing 45 mL of the supernatant and 2.5 mL of the sucrose cushion. Exosomes were then pelleted by diluting the interphase with 50 mL of 1X PBS and ultracentrifuging at 100,000 RCF for 2 h at 4 °C.

For CrPV derived exosomes used in sucrose gradients, Western blot analysis, and proteomic analysis, S2 cells were infected with CrPV for 6 h (MOI 10) by washing the cells with PBS and then incubating with virus at 25 °C for 30 mins. After viral adsorption, cells were washed with PBS followed by addition of exosome-depleted media and incubation at 25 °C. The supernatant was harvested and exosomes were isolated as stated above. For analysis of enveloped CrPV, S2 cells were infected for 24 h (MOI 10) before harvesting the supernatant.

### Sucrose and iodixanol gradients

For density estimation of exosomes from S2 cells, exosomes were first isolated as above and then layered onto an 11 mL linear sucrose gradient (0.25–2 M sucrose). The gradients were then centrifuged at 100,000 RCF for 18 h at 4 °C. Fractions were collected using an ISCO fractionator and density was determined by comparing fractions to a standard curve using a Brix/RI-Check refractometer (Reichart).

To separate exosomes and enveloped CrPV from non-enveloped virus we adapted a protocol that employs isopycnic iodixanol, or Optiprep, gradients (Invitrogen) to separate enveloped from non-enveloped virus^5^. Briefly, cell-culture supernatants were clarified by serial centrifugation at 1000 RCF for 10 minutes at 4 °C, followed by 10,000 RCF for 30 minutes at 4 °C. Virus and exosomes were then pelleted by centrifuging at 100,000 RCF for 1 h at 4 °C. The resulting pellet was resuspended in 500 µL of 1X PBS and layered onto an 8–40% iodixanol step gradient. Gradients were centrifuged at 141,000 RCF in a SW 41 Ti rotor (Beckman) for 48 h at 4 °C. Fractions were collected from the top by hand.

### Protein digestion and duplex demethylation labeling

Cell or exosome pellets harvested from CrPV-infected cells at 6 hpi were solubilized in 6 M urea and 2 M thiourea. Protein concentrations were determined via BCA assay (Thermo). Equal amounts of protein (50 µg) from each sample was reduced by adding 1 µg of dithiothretiol and incubating for 30 min at room temperature. Proteins were alkylated with the addition of 5 µg iodoacetamide and allowed to incubate for 20 min at room temperature. Samples were digested with LysC before dilution with 4 volumes of 50 mM ammonium bicarbonate and digestion overnight with trypsin. Digested peptides were purified and concentrated on C18 STAGE-tips, eluted in 80% acetonitrile, 0.5% acetic acid, and dried in a vacuum concentrator (Eppendorf). Dried peptides were resuspended in 100 mM triethylammonium bicarbonate and chemical demethylation labeling was performed using light (CH_2_O; mock-infected samples) or heavy (^13^CD_2_O; CrPV-infected samples) isotopologues of formaldehyde^20^. Labeled samples were combined and STAGE-tip purified. Eluted samples were dried and resuspended in 20% acetonitrile and 0.1% formic acid.

### Liquid chromatography tandem mass spectrometry (LC-MS/MS) analysis

Digested peptides were analyzed by LC-MS/MS using a nanoflow HPLC (Thermo easy-nLC1000) coupled to a Q-Exactive mass spectrometer (Thermo). For each sample, 5.0 μg of peptides (based on the protein quantitation) were injected into the LC and loaded onto an in-house packed fused-silica (5 μm Aqua C18 particles (Phenomenex)) fritted trap column (2 cm, 100 μm I.D., 360 μm O.D., 5 μL/min flow rate, Buffer A = 0.5% acetic acid), then resolved on a reverse phase 75 μm inner diameter fused silica, in-house packed 30 cm analytical column (ReproSil C18, 3 μm particle size (Dr. Maisch)) using a 75 min linear gradient run at 250 μL/min from 5% to 35% Buffer B (acetonitrile, 0.5% acetic acid), followed by a 15 min wash at 95% Buffer B. Instrument acquisition parameters included a 1% underfill ratio, 70 000 precursor mass resolution, 17 500 fragment mass resolution, normalized collision energy (NCE) of 28%, +1 and unassigned charges were excluded, “exclude isotopes” was turned on, intensity-dependent MS/MS at 1.7e5 intensity threshold, and the instrument was set to scan from 300 to 2000 m/z with a 30 s dynamic exclusion time.

Data were searched using MaxQuant (v1.5.3.30)^21,22^. Parameters included: carbamidomethylated cysteine (fixed), methionine oxidation (variable), glutamine and asparagine deamidation (variable); trypsin specific; maximum 2 missed cleavages; 10 ppm precursor mass tolerance; 0.05 Da fragment mass tolerance; 1% FDR; +1 to +7 charge states; match between runs and re-quantify; and common contaminants were included. Protein groups required a minimum of 1 peptide to be identified and a minimum of 2 peptides for quantification. Both the *Drosophila* and CrPV protein databases used were downloaded from Uniprot (www.uniprot.org; April 23^rd^, 2014). Using Perseus (v1.5.2.6)^23^, contaminants and reverse hits were filtered out, protein ratios were Log_2_ transformed, and ratios were averaged between replicates.

For GO term enrichment analysis, we employed the Gene Score Resampling (GSR) in ErmineJ v3.02, using the Log2 transformed protein ratios for “protein score”^24^. We considered a GO term to be significantly enriched if the Bejamini Hochberg-corrected GSR p-value was less than 0.05. The mass spectrometry proteomics data have been deposited to the ProteomeXchange Consortium via the PRIDE^1^ partner repository with the dataset identifier PXD011271.

### RT-PCR

RNA from whole cells was extracted using TriZol reagent (Invitrogen). Alternatively, RNA was isolated from gradient fractions using phenol:chloroform extraction. Briefly, each fraction was brought up to 250 µL with RNase-free H_2_O. An equal amount of phenol was then added to each fraction, samples were vortex and centrifuged at 4 °C at 13.2 K RPM for 30 minutes. The aqueous layer was removed and placed into a new tube with and equal volume of chloroform. The sample was vortexed and centrifuged for 5 minutes at 4 °C at 13.2 K RPM. The aqueous layer was once again removed and an equal volume of 100% ispropanol was added in addition to 400 mM of ammonium acetate. Samples were incubated at −20 °C for at least 2 h. RNA was then pelleted by centrifugation for 10 minutes at 13.2 K RPM. The RNA pellet was washed 3 times with 75% ethanol before resuspending in 10 µL RNase-free H_2_O.

For RT-PCR on gradient fractions, the entirety of the extracted RNA was used and 100 ng of *in vitro* synthesized firefly luciferase RNA was added as a standard to each fraction. RT-PCR was performed using Superscript Reverse Transcriptase III (Invitrogen) at 50 °C using a random hexamer primer. CrPV cDNA was amplified using primers P1 (5′ – TCCTCAAGCCATGTGTATAGGA – 3′) and P2 (5′ – GTGGCTGAAATACTATCTCTGG – 3′) while FLuc cDNA was amplified using primers P3 (5′ – ATGAACGTGAATTGCTCAAC – 3′) and P4 (5′ – CCGGATTGTTTACATAACC – 3′).

### Western blots

For cell and exosomal lysates, equal amounts of protein (10 µg) were resolved on a 12% SDS-PAGE gel and then transferred to a polyvinylidene difluoride Immobilon-FL membrane (Millipore). Membranes were blocked for 30 min at room temperature with 5% skim milk in TBST. Blots were incubated for 1 h at room temperature with the following antibodies: CrPV ORF1 (raised against CrPV RdRp) rabbit polyclonal (1:10,000), CrPV ORF2 (raised against CrPV VP2) rabbit polyclonal (1:10,000), mouse anti-syntaxin 1 A (1:5000; DSHB), mouse anti-ubiquitin (1:5000; AbCam), or mouse anti-Actin (1:5000; AbCam). Membranes were washed 3 times with TBST and incubated with either goat anti-rabbit IgG HRP (1:20,000; GE Healthcare) or goat anti-mouse IgG HRP (1:5000; Santa Cruz) for 1h at room temperature. Enhanced chemiluminescence (Thermo) with was used for detection.

For westerns performed on gradient fractions, proteins were first extracted using trichloroacetic acid (TCA). To each fraction an equal amount of H_2_O was added for a total volume of 400 µL. 100 µL of 100% TCA was added and samples were incubated on ice for 10 minutes. Protein was pelleted by centrifugation for 10 minutes at 4 °C at 16.1 RCF. Supernatant was removed and the pellet was washed twice with 200 µL of ice cold acetone. The final pellet was then dried by incubating at 95 °C for 5 minutes and protein was resuspended in 20 µL of SDS-PAGE loading buffer.

### Electron microscopy

Negative stained specimens were prepared by adsorbing samples to glow discharged carbon-coated copper grids and subsequently staining with uranyl formate. A Tecnai Spirit transmission electron microscope (FEI) operated at an accelerating voltage of 120 kV was used to examine these specimens. Images were acquired using a 4 K × 4 K Eagle charge-coupled device (CCD) camera (FEI) at a nominal magnification of 49,000×.

### Membrane integrity assay

S2 cells (2.5 × 10^6^) were seeded onto 6-well plates. Cells were washed with PBS and infected with CrPV at an MOI of 10 by incubating at 25 °C for 30 min. Cells were then washed with PBS before adding complete Shields and Sang media. At each time point (2, 4, 6, 8 h) cells were harvested and 10 µL of the cell suspension was incubated with an equal volume of trypan blue. Membrane permeability was assessed via trypan blue exclusion using a Countess II Automated Cell Counter (Thermo Fisher). In parallel, the supernatant from each time point was used for viral titre determination as described above. Light micrographs of cells were acquired using a Olympus CKX41 microscope at 40X magnification mounted with a Olympus SC30 camera.

### Treatment of cells with ELVs

CrPV and ELVs were first separated via iodixanol gradients as described above. Fractions corresponding to either ELVs or CrPV virions were pooled and dialyzed in PBS using a Slide-A-Lyzer 10,000 MWCO dialysis cassette (Thermo Fisher). After dialysis, pooled fraction sets were added to S2 cells seeded in a 6-well plate without media and incubated for 1 h at 25 °C. Following incubation, cells were washed with PBS and complete S2 cell media was added. Cells were incubated at 25 °C for 48 h before being harvested for viral yield determination.

## Results

### Isolation of exosome-like vesicles from S2 cells during CrPV infection

**Figure 1:**
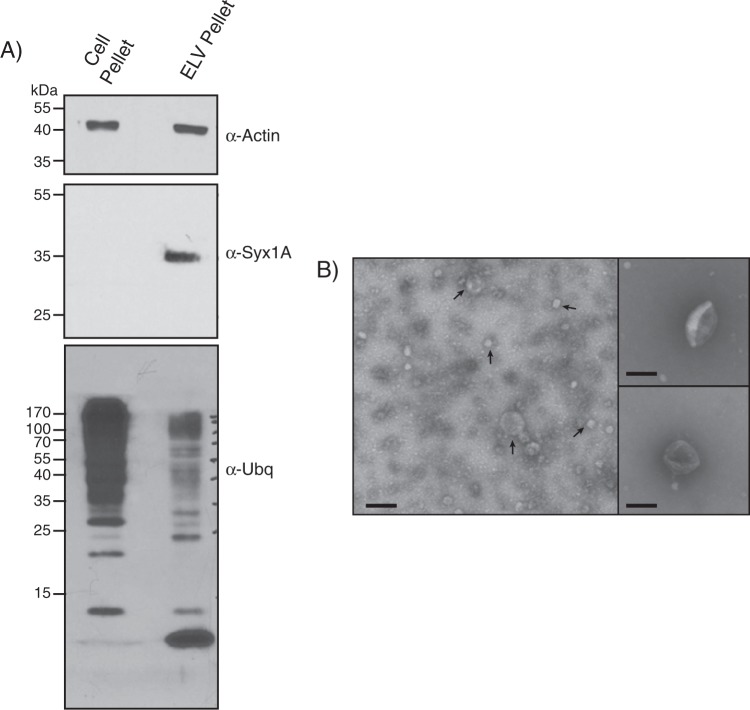
Validation of exosome-like vesicles isolated from Drosophila S2 cells. (**A**) Western blot analysis of ELV markers in the cell pellet and ELV pellet after isolation. Syx1 A = Syntaxin-1A; Ubq = Ubiquitin. (**B**) Transmission electron micrograph of ELVs isolated using differential ultracentrifugation. Micrographs shown are from a 1:2 dilution (left panel) or 1:50 dilution (right panels) in PBS before TEM. Scale bar represent 100 nm. Uncropped gel images are shown in Supplemental Fig. 1.

To characterize exosome-like vesicles (ELVs) under CrPV infection, we isolated ELVs from *Drosophila* S2 cells that were mock- or CrPV-infected using differential ultracentrifugation as previously described^13^. Both the S2 whole cell pellet and the ELV pellet were analyzed by Western blot to assess the presence of exosomal protein markers (Fig. 1A)^25^. As reported previously^13^, both pellets contained β-actin, and ubiquitin. Ubiquitin is thought to aid in protein sorting to exosomes on the cytosolic side of the multi-vesicular body, therefore an enrichment of specific proteins would be expected in the ELV pellet^26^. Using an anti-ubiquitin antibody, differential banding patterns were observed between the cell pellet and ELV fraction. (Fig. 1A). Moreover, syntaxin-1A showed a strong enrichment in the ELV fraction compared to the cell pellet as observed previously (Fig. 1A)^13,25^. In addition to Western blot analysis, we employed negative stain electron microscopy to assess the integrity of the vesicles. Electron micrographs of the vesicles displayed a range in size of 30–100 nm, typical of exosomes isolated from mammalian cells (Fig. 1B)^27^. As reported^13,25^, these results indicate that *bona fide* exosome-like vesicles can be isolated from S2 cells.

**Figure 2:**
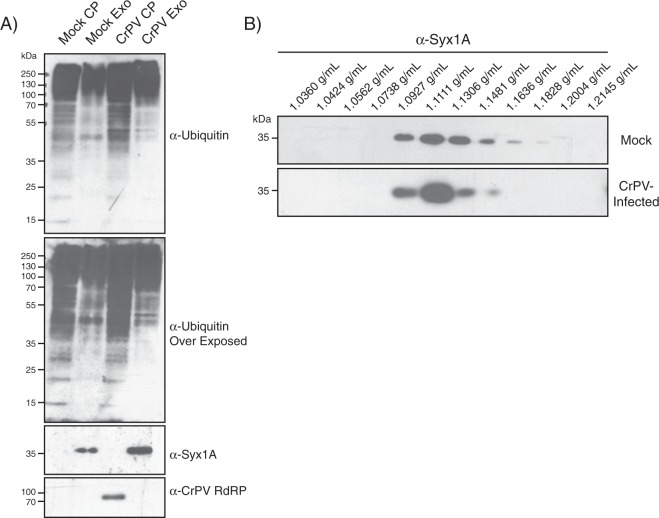
Isolation of Exosome-like vesicles from Drosophila S2 cells during CrPV infection. (**A**) Western blot analysis of the cell pellet (CP) versus the ELV pellet (Exo) in either mock- or CrPV-infected S2 cells. Cells were infected at an MOI of 10 and exosomes were harvested at 6 hours post infection. Syx1 A = Syntaxin-1A; Ubq = Ubiquitin. (**B**) Sucrose density gradient separation of exosome-like vesicles isolated from mock- or CrPV-infected cells at 6 hpi. Uncropped gel images are shown in Supplemental Figs 2, 3.

We next investigated ELVs from S2 cells infected with CrPV. We chose to infect cells for 6 h to mitigate contamination of the ELVs by cellular debris due to lysis^28,29^. Similar to ELVs from mock-infected cells, syntaxin-1A was enriched in the ELV fraction compared to the cell pellet (Fig. 2). Interestingly, distinct ubiquitylated proteins could be detected when comparing ELVs isolated from mock and CrPV-infected cells suggesting that there may be changes in protein content and/or ubiquitylation of proteins in the vesicles (Fig. 2A). Using an antibody that recognizes the RNA-dependent RNA polymerase (RdRP) of CrPV, CrPV RdRP was present in the CrPV-infected cell pellets, but not in the ELV fractions suggesting that it is not packaged into ELVs.

To further validate that exosome-like vesicles were isolated, we measured their density via sucrose gradients. ELV pellets were isolated and then layered onto a 0.25–2 M sucrose density gradient. After centrifugation, the gradients were fractionated and the distribution of syntaxin-1A across the gradient was monitored by immunoblotting (Fig. 2B). From both mock- or CrPV-infected cells, syntaxin-1A was present in fractions of a density range from 1.09–1.16 g/mL, consistent with the range of ELVs^9^ (Fig. 2B). Altogether these results demonstrate that exosome-like vesicles were isolated from CrPV-infected cells.

### Proteomic analyses of CrPV-infected cells and derivative ELVs

**Figure 3:**
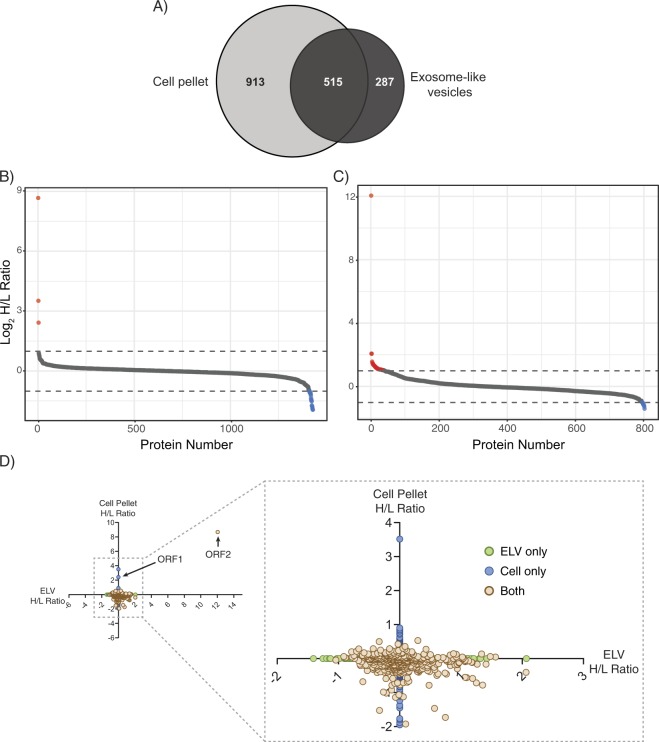
Quantitative proteomic comparison of cell pellets and ELVs isolated from mock- and CrPV-infected cells at 6 hours post infection. (**A**) Venn diagram of proteins identified in S2 cell pellets and ELVs from both mock- and CrPV-infected cells. Cells and ELVs were collected from both mock and CrPV-infected S2 cells at 6 hpi. Proteins were collected from all samples and equal amounts were subjected to trypsin digestion. Peptides from both the cell pellet and ELV pellets were dimethylated labeled with light formaldehyde (L; mock-infected) or heavy formaldehyde (H; CrPV-infected). Peptides were then pooled and analyzed by LC-MS/MS. (**B**) and (**C**) Log_2_ abundance ratio distribution of proteins quantified from CrPV- and mock-infected cells for the (**B**) cell pellets and the (**C**) ELVs. (**D**) Scatter plot of proteins quantified in S2 cell pellets in comparison to those in ELVs. Plotted is the Log_2_ transformed abundance ratios comparing the heavy (CrPV-infected) to light (mock) labeled samples (H/L ratio). Boxed region is zoomed-in area of the graph lacking viral proteins.

Our Western blot analyses hinted that the protein composition of ELVs is different between mock- and CrPV-infected S2 cells. To address this, we utilized quantitative mass spectrometry to identify changes in the proteomes of ELVs (Fig. S4). Proteins of ELVs from mock- or CrPV-infected S2 cells (6 h) were isolated and digested with trypsin to produce peptides that were subsequently labeled with a ‘light’ formaldehyde reagent (mock-infected) or ‘heavy’ formaldehyde reagent (CrPV-infected) before analyzing via LC-MS/MS (Fig. S4)^20^. This analysis was also performed on cell pellets in parallel (with the same labeling procedure) to determine if proteins enriched in ELVs was due to an increased abundance within the cell or specific packaging of host proteins. From this analysis, we identified and quantified 1428 and 802 proteins from the cell and ELV pellets, respectively, in 2 of 3 biological replicates (Fig. 3). Of these, 913 and 287 proteins were specific to the cell and ELV pellets, respectively, while 515 proteins were shared between both (Fig. 3A). For proteins quantified in the cell pellet, only 3 displayed a >2-fold increase in protein abundance in CrPV-infected compared to mock-infected cells (red points; Fig. 3B). These included both viral polyproteins (ORF1 and ORF2) and the uncharacterized protein, CG31731-RC (Fig. 3B and D). The majority of proteins quantified in the cell pellet at 6 hpi decreased in protein abundance with 16 proteins being down-regulated more than 2-fold compared to mock-infected cells (blue points; Fig. 3B). These results are consistent with the observation that overall host translation is shut down in CrPV-infected cells concomitant with increased viral protein synthesis^19,30^. In the ELV pellet, 40 proteins increased >2-fold in abundance at 6 hpi while 8 proteins decreased >2-fold (red and blue points, respectively; Fig. 3C).

**Figure 4:**
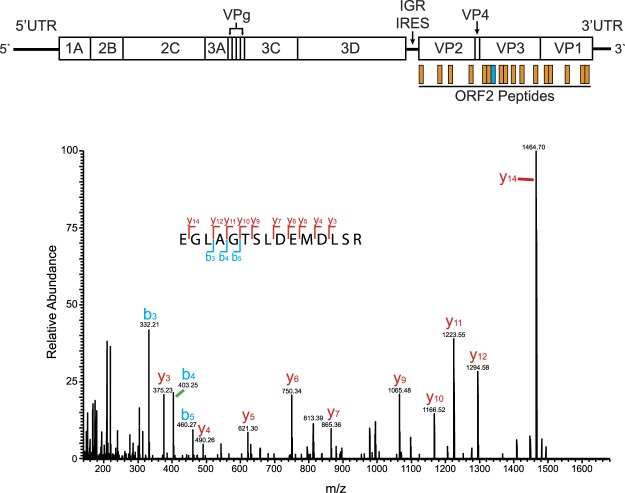
CrPV ORF2 peptides identified in ELVs from CrPV-infected S2 cells. Top: schematic displaying 21 peptides mapped to CrPV ORF2 (note: does not include peptides with missed cleavages). Bottom: MS/MS spectra of one peptide identified (blue in schematic) from the CrPV ORF2 enriched in ELVs derived from CrPV-infected cells. Fragment spectra from the 813.40 m/z doubly charged, formaldehyde labeled (+32 Da) precursor ion of EGLAGTSLDEMDLSR from trypsin digested ELVs. In total, 21 peptides were mapped to CrPV ORF2. Blue bar indicates location of displayed peptide. Individual fragment ions are annotated in the spectrum and in the sequence representation. In red are y-ions, while b-ions are represented in blue.

We also identified multiple markers that have been associated with exosomes in the ELV pellet such as ALIX, Syntaxin-1A, Rab35, and Flotillin-1, further supporting that *bona fide* exosomes were isolated^13^. Surprisingly, CrPV ORF2 peptides were found in the ELV pellet (Log_2_ ratio_ = _12.04; Figs 3D and 4, and Table S3). In contrast, there were no peptides mapped to the ORF1 of CrPV in the ELV pellet; congruent with our previous Western blot analysis (Figs 2, 3D, and Tables S1 and S2).

To gain further insight into the cellular processes underlying proteins in the cell pellet and ELVs in CrPV-infected cells, we performed a gene score resampling (GSR) analysis. Unsurprisingly, there were no significantly enriched terms found within the cell pellet protein list since CrPV infection results in global translation shut down. On the other hand, many GO terms were significantly enriched (Benjimini Hochberg-corrected p-value < 0.05) in ELVs, such as those linked to transport (e.g. GO:0006818; GO:0006811; GO:0055085) and metabolic processes (e.g. GO:0006091; GO:0006793; GO:0051186) (Table S1). Given that biogenesis of ELVs occurs through invagination of the endosomal membrane, it is unsurprising that a large portion of proteins are annotated with metabolic functions as these processes largely occur in the cytoplasm. Altogether, these results suggest that the protein composition of ELVs during CrPV-infection is unique compared to that of the cell pellet and ELVs derived from mock-infected cells.

### CrPV may hijack ELVs during infection

**Figure 5:**
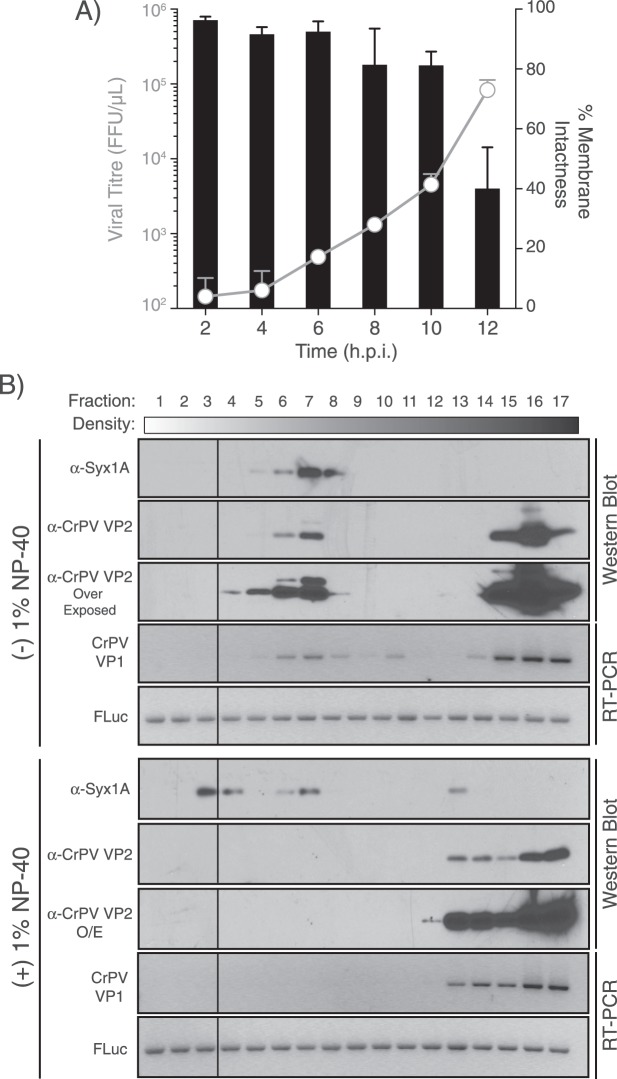
ELVs from CrPV-infected S2 cells contain CrPV RNA and structural protein. (**A**) Membrane integrity and extracellular viral yield in CrPV-infected S2 cells. S2 cells were infected with CrPV at an MOI of 10 and viral loads were measured in the supernatant at various time points throughout infection via a fluorescence foci forming assay (open circles). Exclusion of trypan blue staining of S2 cells (i.e. percent cell viability) was used as a proxy for membrane integrity at each time point (bar graph). Shown is the average from three biological replicates (±SD). (**B**) Iodixanol gradient analysis of ELVs from mock- and CrPV-infected S2 cells. ELVs from CrPV-infected S2 cells (MOI 10, 24 hpi) were treated with or without 1% NP-40 and layered onto an 8–40% iodixanol gradient and centrifuged at 141,000 g for 48 hours. Fractions were collected, split, and protein was extracted via trichloroacetic acid precipitation and RNA via phenol-chloroform. Syx-1A was used as a marker for ELVs. Equal amounts of *In vitro* synthesized firefly luciferase RNA was doped into each fraction to control for RT-PCR efficiency. Shown is a representative gel from three independent experiments. Uncropped gel images are shown in Supplemental Fig. S5.

The observation that ORF2 (i.e. structural), but not ORF1 proteins from CrPV are found in ELVs suggests that CrPV may commandeer these vesicles during infection. To rule out that the resulting CrPV-containing ELVs are a result of apoptosis/lysis from infected S2 cells, we examined the extracellular viral titres and integrity of the cell membrane by trypan blue exclusion assay over the course of an 8 h viral infection (Fig. 5A). Increasing extracellular virus is detected throughout the course of infection with an increase at 6 hpi and increases substantially onwards (Fig. 5A). By 8 hpi, ten times more virus is detected in the supernatant relative to that at 2 hpi (Fig. 5A). Interestingly, the membrane of S2 cells remains intact up until 12 hpi, suggesting that the virus being released into the supernatant is not due to overt cell lysis. This is corroborated through examination of infected cells under light microscopy (Fig. S6). Minimal cytopathic effects are visible (i.e. lifting off the substratum) up until 10 hpi and onwards where distinct cell lysis is observed (Fig. S6; black arrows).

It is possible that CrPV virions are associated with the ELVs and not necessarily within the vesicles. To distinguish these possibilities, we performed iodixanol gradients on the extracellular contents of CrPV-infected S2 cells. Iodixanol gradients are sensitive to changes in sample densities and thus exosomes and virions fractionate differentially^31^. From CrPV-infected S2 cells, supernatants were collected and ELVs (and virus) were pelleted via ultracentrifugation before layering onto an 8–40% iodixanol gradient. The gradient was subsequently fractionated and analyzed by Western blot and RT-PCR analyses for the presence of viral proteins or RNA, respectively. Consistent with our mass spectrometry data, CrPV VP2 structural protein had a bimodal distribution, with one population that co-fractionates with syntaxin-1A, which represents ELVs and one that sediments to the bottom of the gradient (Fig. 5B), the latter of which likely represents fully assembled virions containing viral RNA. For RT-PCR analysis, equal amounts of a control *in vitro* transcribed luciferase RNA were aliquoted into each fraction, which was used as a RT-PCR normalization control across the gradient. The CrPV RNA exhibited a bimodal distribution that co-sedimented similarly to the VP2 protein, thus suggesting that both viral RNA and protein are within the ELV fractions. To determine if the syntaxin-1A-containing fractions include virions enveloped with a lipid, we treated isolated ELV pellets with membrane-disrupting NP-40 detergent before layering onto the iodixanol gradients. Comparing to untreated ELVs, NP-40-treated ELVs resulted in a broad distribution of syntaxin 1 A, indicating that the lipid envelope of ELVs was disrupted. Furthermore, NP-40 treatment abolished the bimodal distribution of CrPV VP2 and RNA resulting in an enriched population near the bottom of the gradient, strongly suggesting that the CrPV structural proteins and RNA are enveloped with a membrane within ELVs (Fig. 5B).

**Figure 6:**
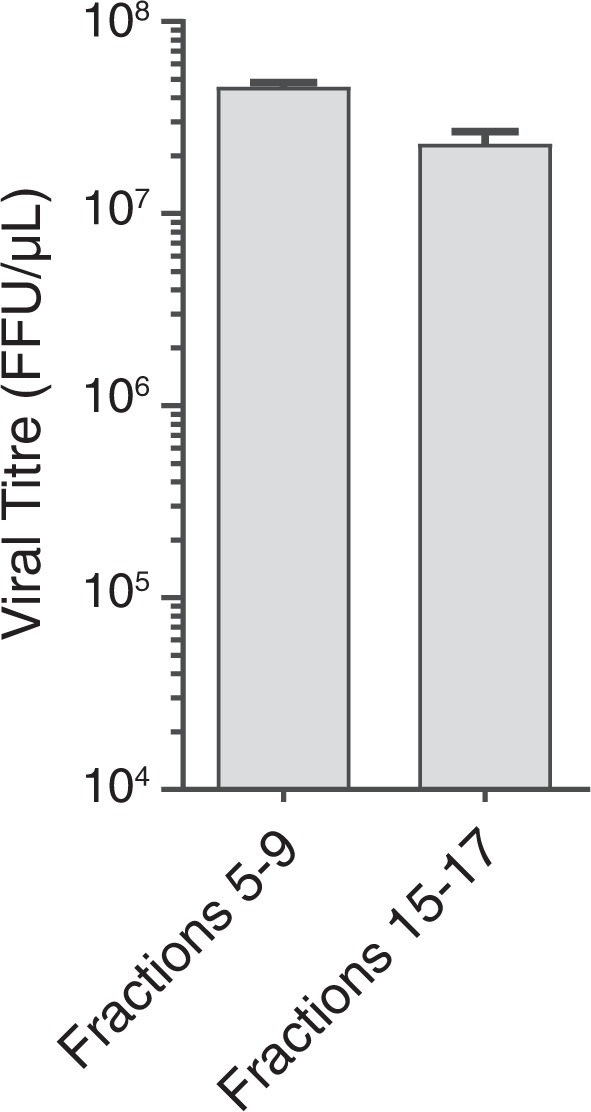
ELVs from infected S2 cells are infectious. ELVs from CrPV-infected cells (24 hpi) were isolated and subjected to iodixanol gradients analysis. Fractions 5–9 (ELVs) and 15–17 (virions) were collected, pooled, and added to naïve S2 cells. After 48 hours, cells were lysed and viral titres were determined using a fluorescence foci forming assay. Shown is the average from three biological replicates (±SD).

We next determined whether the light and dense fractions containing CrPV protein/RNA are infectious. Pooled fractions containing ‘naked’ CrPV (fractions 15–17) or ‘enveloped’ CrPV (eCrPV; fractions 5–9) were dialyzed with PBS and then incubated with S2 cells for 48 h (Fig. 6A). Supernatants from cells incubated with either CrPV or eCrPV resulted in an approximately equal amount of viral titre after 48 h, suggesting that both CrPV and eCrPV fractions are infectious (Fig. 6). Taken together, these data suggest that CrPV can commandeer ELVs to facilitate infection in *Drosophila* cells.

## Discussion

Viruses have evolved novel strategies to evade the host immune system. In recent years, it has been demonstrated that non-enveloped viruses have the ability to co-opt cellular processes to acquire an envelope^32,33^. In this study, we provide evidence that CrPV structural proteins and viral RNA are enveloped within ELVs derived from CrPV-infected S2 cells and that these ELVs are infectious. By using proteomic approaches, we identified 1408 and 802 proteins from the cell and exosome-like vesicles, respectively (Fig. 3). Only 64% of the ELV-associated proteins overlapped with the cell pellet suggesting that there may be a distinct set of proteins enriched within ELVs compared to the cell pellet. Moreover, during CrPV infection, 40 proteins increased and 8 protein decreased in abundance compared to mock-infected cells (Fig. 3), thus suggesting an active mechanism for sorting and secretion of proteins in infected-S2 cells. We propose that CrPV uses both lytic and non-lytic egress strategies as part of its viral life cycle.

The best-described mechanism for the formation of exosomes in mammalian cells is through the endosomal sorting complex required for transport (ESCRT)^27^. This complex is composed of roughly thirty proteins that assemble into four complexes (ESCRT-0, -I, -II, and –III) along with other associated proteins. ESCRT-0 binds and sequesters ubiquitylated transmembrane proteins in the endosomal membrane. HRS (a component of ESCRT-0) then recruits TSG101 of the ESCRT-I complex. In tandem with ESCRT-II, ESCRT-I acts to cause endosomal membrane deformation. ESCRT-I is then involved in the recruitment of ESCRT-III via ESCRT-II or ALIX. Finally, ESCRT-III facilitates vesicle scission into the endosomal lumen^27,34^. The exact mechanism of how cytosolic proteins are sorted into exosomes is not well understood. It is thought that mono-ubquitinylated proteins containing ‘late’ domain with a sequence motif P(S/T)XP, YPX_1-3_L, or PPXYY are targeted for delivery to MVBs by interacting with proteins such as TSG101 or ALIX^26,32,35^. Alternatively, a recent role for HSC70 has been proposed where it binds proteins containing a KFERQ sequence and to phosphatidylserine on the MVB outer membrane^36^. However, not all proteins in exosomes are ubquitylated nor do they all contain KFERQ sequences. It appears a passive mechanism may be involved in protein sorting to MVBs that involves lipid (e.g. ceramide or cholesterol) and/or tetraspannin associations^37–40^. Currently there is limited knowledge on how CrPV alters host cell processes during infection and although numerous other viruses have been shown to exploit exosome biogenesis pathway including human immunodeficiency virus-1 (HIV-1), hepatitis B virus (HBV), and Epstein Barr virus (EBV), there remains no consensus view of a distinct mechanism that leads to changes in cargo content of exosomes^41^.

From our proteomic analysis, the only protein to be enriched outside of viral proteins in the cell pellet was CG31731-RC, also recently termed Eato^42^. This protein is homologous to the *C. elegans* CED-7 and mammalian ABCA1 proteins and has recently been shown to be important for the cellular engulfment pathway in *Drosophila* ovaries^42^. In mouse hemocytes and thymocytes, ABCA1 has a role in presentation of phosphatidylserine after apoptotic stimuli, and thus may act in dying cells to promote phagocytic engulfment^43^. Moreover, CED-7 in *C. elegans* has been shown to be important for the presence of extracellular vesicles containing engulfment signals and its activity is required in both phagocytic and dying cells for engulfment^44,45^. Given that phagocytosis plays an important role in *Drosophila* innate immunity^46^, upregulation of CG31731/Eato may act as first line defense against viral infection to clear infected cells. Alternatively, as discussed below, this protein may be important in secretion of anti-viral vesicles involved in the RNAi response^16^. Further study of this protein and its role during infection will likely provide insight into the host-virus interactions occurring in *Drosophilia*.

Surprisingly, the most highly enriched protein(s) in ELVs derived from CrPV-infected cells was the CrPV structural proteins. We identified 21 peptides that mapped across the CrPV ORF2 polyprotein resulting in a log_2_ fold change of >12 in ELVs (Figs 3 and 4). Interestingly, we were unable to identify any of the CrPV non-structural proteins encoded by ORF1 either by mass spectrometry or Western blot within the ELV fraction (Figs 2 and 3). Together with our density gradient analysis, viral titres, and membrane integrity assay (Figs 5 and 6), these results indicate that, like hepatitis A virus (HAV) and hepatitis E virus (HEV), CrPV may co-opt exosomes for its own advantage during infection^32^. Akin to our observations with CrPV, HAV infection leads to two distinct virus populations of virus; both populations remain infectious, however one acquires an envelope (eHAV) while the other remains non-enveloped^5^. HAV encodes two tandem ‘late’ domains in its VP2 capsid protein that are proposed to facilitate interaction of the viral capsid with ALIX and be subsequently sorted into exosomes. Intriguingly, eHAV contains an unprocessed version of the viral capsid protein VP1, termed VP1pX, that is not present in the non-enveloped virus suggesting there could an additional role played by the pX extension to facilitate envelopment of HAV^5^. Similarly, HEV also contains a viral protein (ORF3) that is only found in enveloped forms of the virus which directs interacts with TSG101 through a PSAP motif^47,48^. Whether CrPV follows a similar mechanism(s) remains to be determined. Further studies investigating the role of the ESCRT complex and how viral proteins contribute to this process in CrPV infection would provide valuable insights into its mode of transmission.

Why would CrPV require an envelope for cell-to-cell transmission? With HAV, enveloped virions circulate in the blood of infected patients while non-enveloped virions are found in the feces^5^. For decades virologists have understood that enveloped and non-enveloped viruses have distinct advantages over on another. Enveloped viruses can readily manipulate the contents of their cognate envelope allowing for flexibility in receptors, evasion of the host immune response, and shuttling viral proteins to enhance infection. Nevertheless, envelopes are typically susceptible to bile salts and retain lower stability in the environment (e.g. due to desiccation or chemical resistance)^49^. Thus, a biphasic viral life cycle provides the security of an envelope with the robustness of a capsid. In the case of eHAV, the envelope cloaks the virus from neutralizing antibodies in the bloodstream. Unlike humans, *Drosophila* lacks an adaptive immune system; however, hemocytes circulating in the hemolymph mediate phagocytosis of invading pathogens to prevent infection^46^. Furthermore, *Drosophilia* cells secrete the Ig-domain containing protein, Dscam, which can be expressed as over 18,000 isoforms and may act in opsonizing pathogens for phagocytosis^46,50^. It is plausible that CrPV hijacks the exosomal pathway to shroud itself from the phagocytic machinery within the host organism. Finally, insect ELVs are not only pro-viral; ELVs secreted from hemocytes of virally-infected fruit flies contain virally-encoded siRNAs to elicit a systemic RNAi immune response^16^. It will be important to determine the mechanisms by which host and viral determinants govern pro- vs anti-viral ELVs and whether the ELVs are more infectious than non-enveloped CrPV.

Overall, our data offers the first look into how extracellular vesicles contribute to the pathogenesis of CrPV and how these viruses are transmitted between host cells. Moreover, it suggests that this virus can co-opt these vesicles for its own advantage to potentially evade the host immune response. Whether other members of dicistroviruses utilize ELVs and how this process is regulated during dicistrovirus infection warrants further investigation.

## Electronic supplementary material

Supplemental Information
