# Supplementary material for: Transmission of Cricket paralysis virus via exosome-like vesicles during infection of Drosophila cells
Source: Sci Rep. 2018 Nov 26;8:17353. doi: 10.1038/s41598-018-35717-5 (PMC6255767; doi:10.1038/s41598-018-35717-5)
Supplement: Supplementary file 1 — Supplemental Information [file 41598_2018_35717_MOESM1_ESM.pdf]

Transmission of *Cricket paralysis virus* via exosome-like vesicles during infection of *Drosophila* cells

Craig H Kerr[1,2], Udit Dalwadi[1], Nichollas E Scott[3], Calvin K Yip[1], Leonard J Foster*[1,2], and Eric Jan*[1]

[1]Department of Biochemistry and Molecular Biology, [2]Michael Smith Laboratories, University of British Columbia, Vancouver BC, V6T 1Z3, [3]Department of Microbiology and Immunology, University of Melbourne, Melbourne, Australia

*corresponding authors: foster@msl.ubc.ca and ej@mail.ubc.ca

SUPPLEMENTARY FIGURE LEGENDS

Supplemental Figure 1. Uncropped gel images of those seen in Figure 1.

Supplemental Figure 2. Uncropped images of those seen in Figure 2A.

Supplemental Figure 3. Uncropped images of those seen in Figure 2B.

Supplemental Figure 4. Schematic of the dimethyl labeling approach for quantitative proteomics. Briefly, mock- or CrPV-infected S2 cells were harvested and exosome-like vesicles were isolated from each respective condition. Proteins were isolated and subjected to trypsin digestion. Peptides were subsequently labeled with a ‘light’ formaldehyde reagent (mock) or ‘heavy’ formaldehyde reagent (CrPV-infected). Peptides were then pooled between either ELV samples or cell pellet samples and subjected to LC-MS/MS analysis. See Materials and Methods for a more detailed description.

Supplemental Figure 5. Uncropped images of those seen in Figure 5B.

Supplemental Figure 6. Light micrographs of CrPV infected cells at various time points. Micrographs were imaged using at 40X magnification with an Olympus SC30 camera mounted on an Olympus CKX41 microscope. Shown are representative fields of view.

Supplemental Figure 1

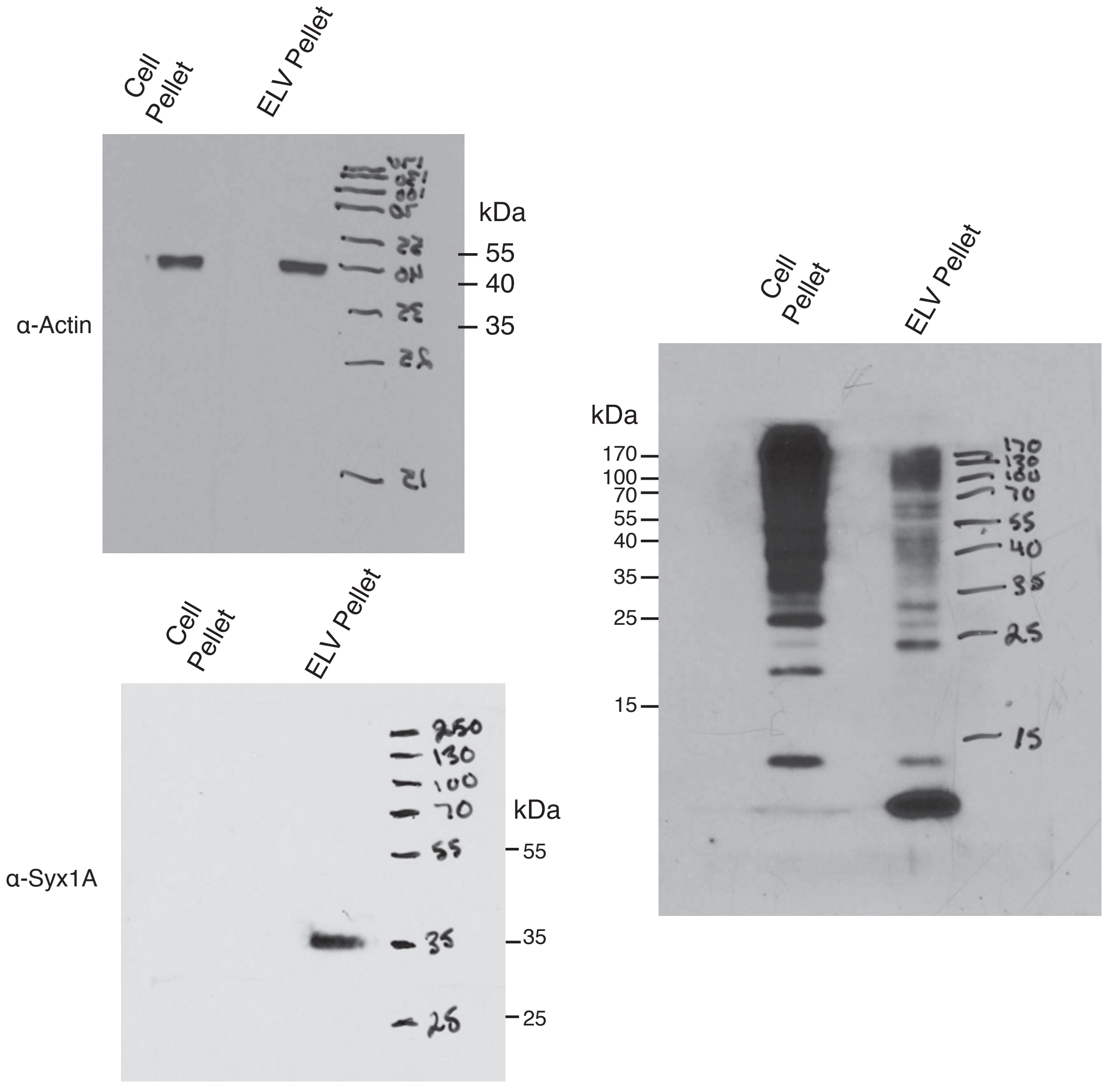

Supplemental Figure 2

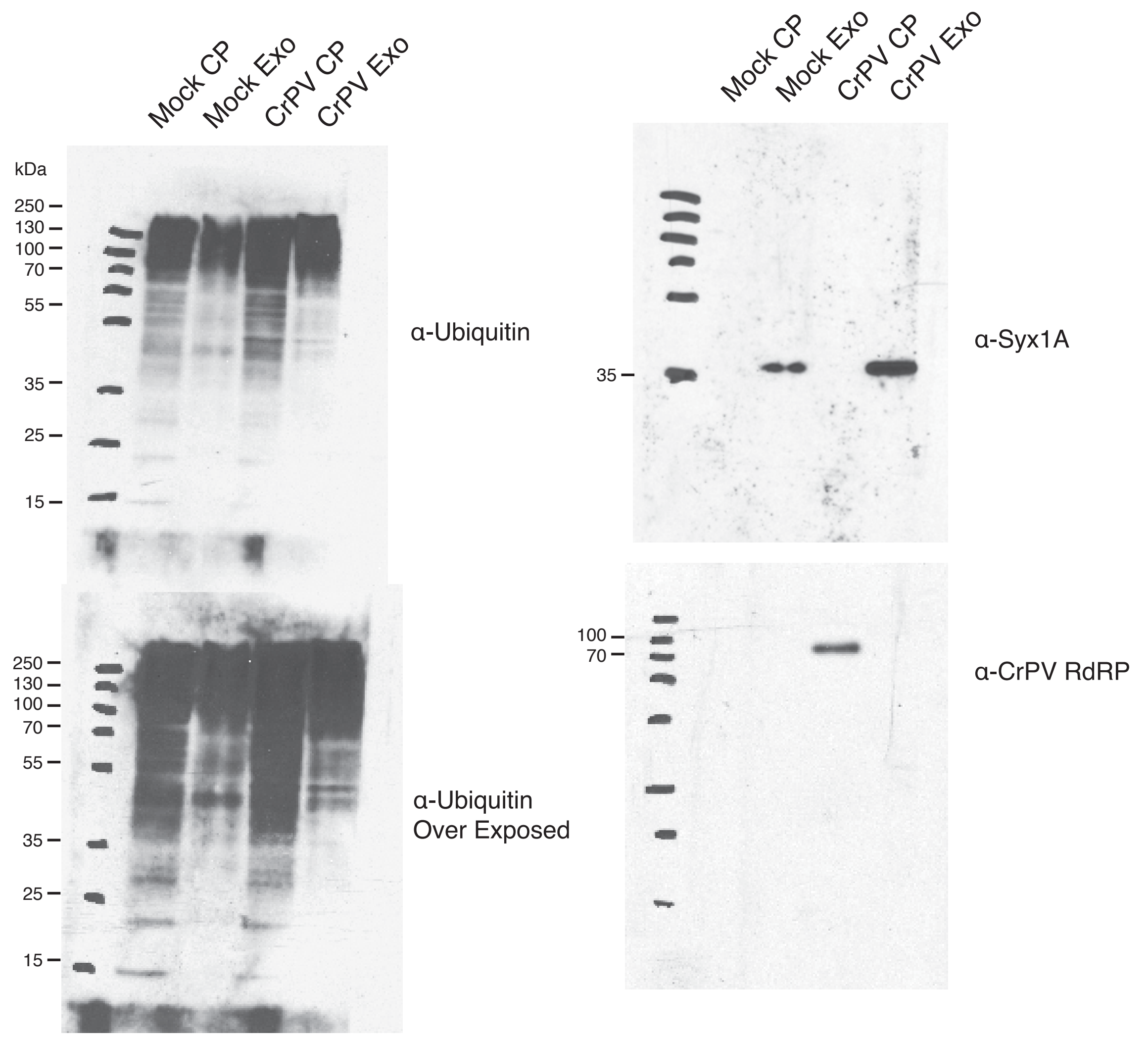

Supplemental Figure 3

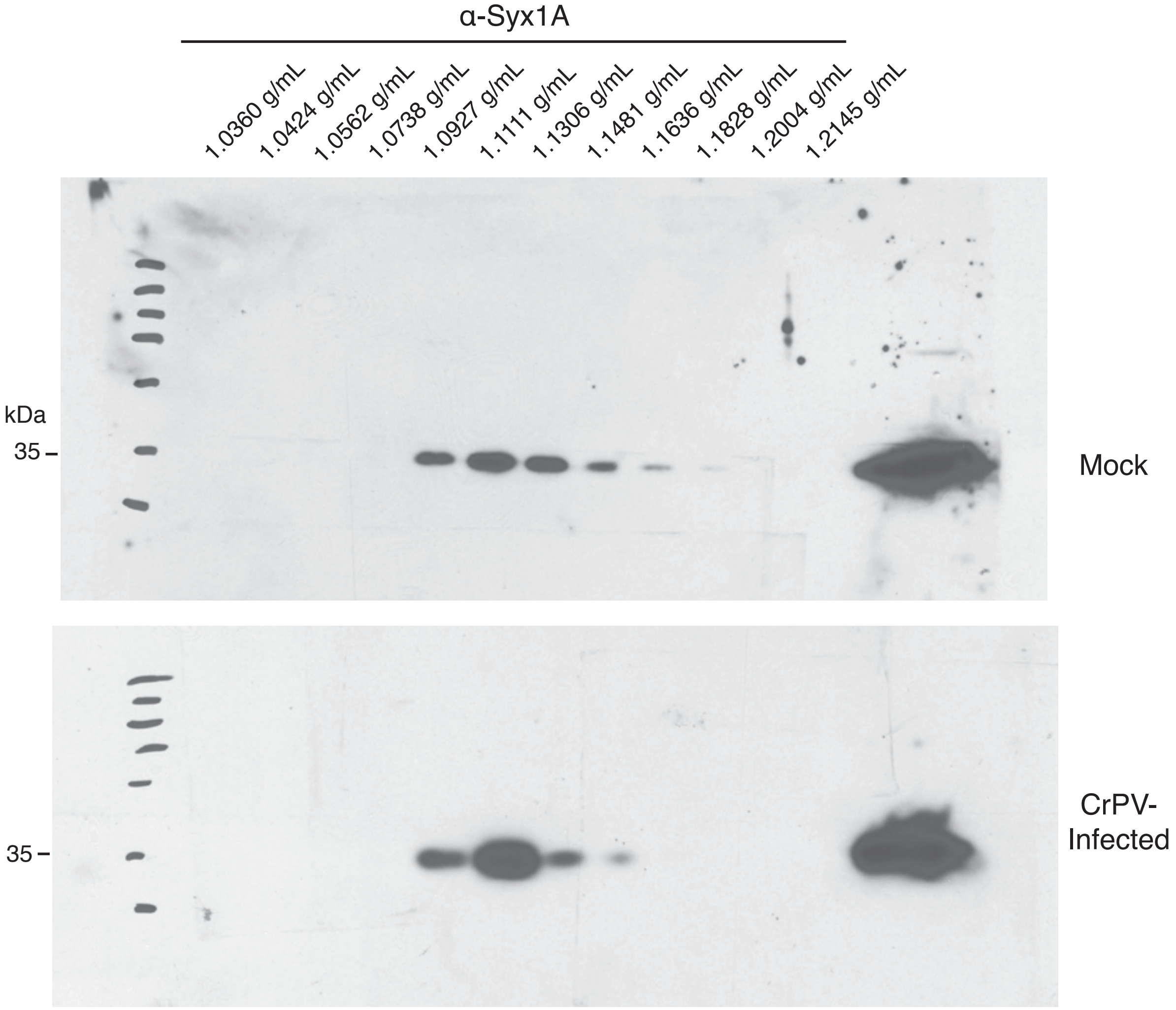

Supplemental Figure 4

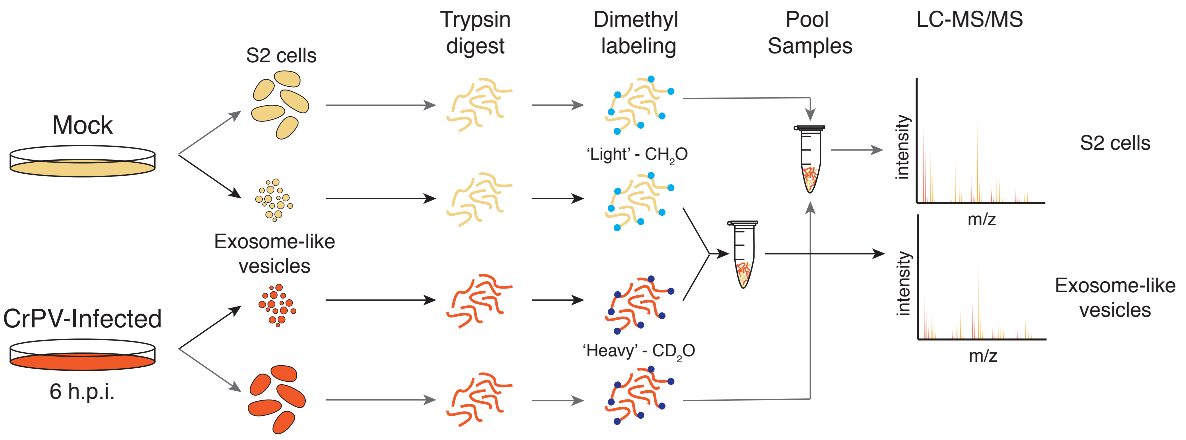

Supplemental Figure 5

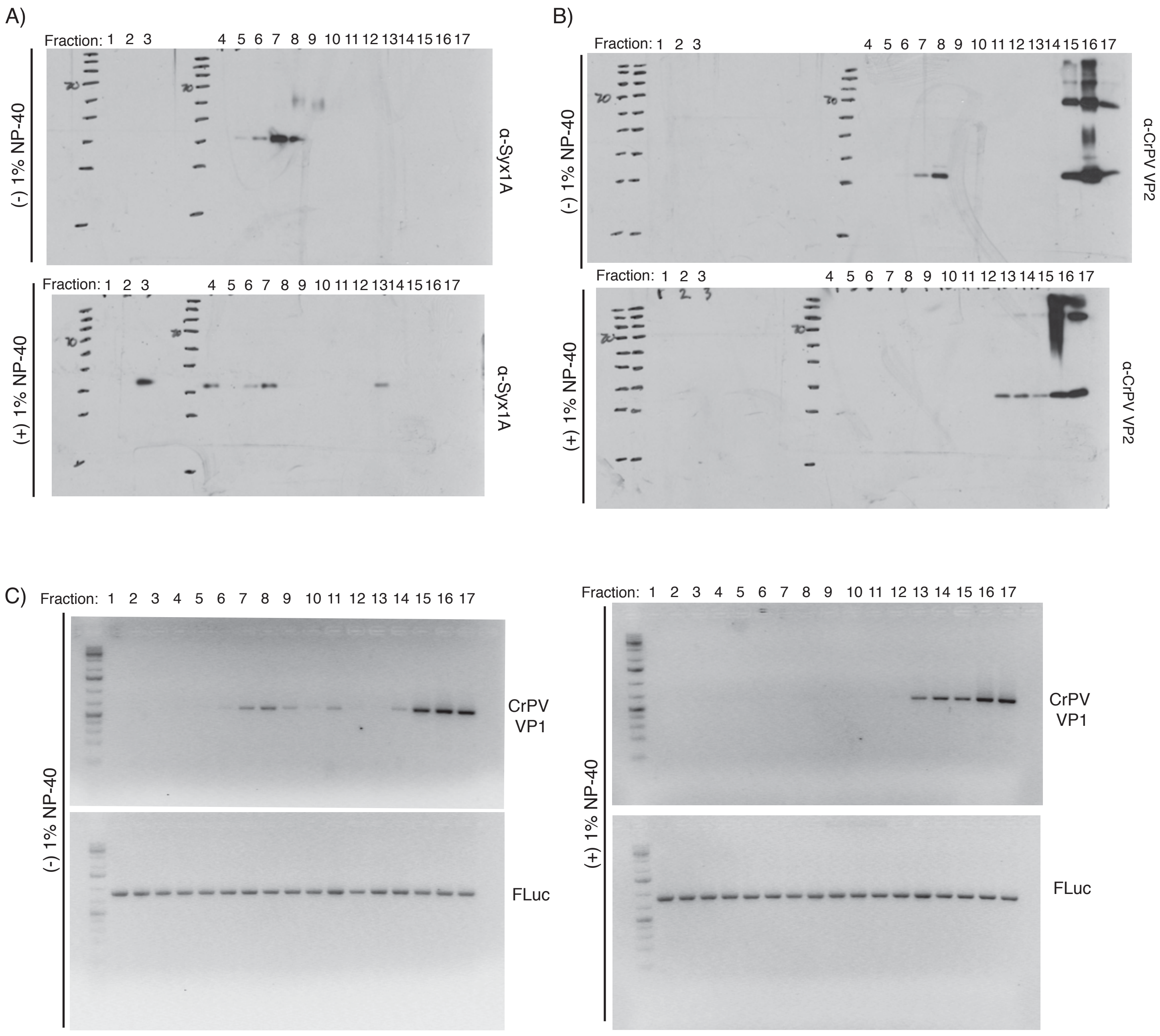

Supplemental Figure 6

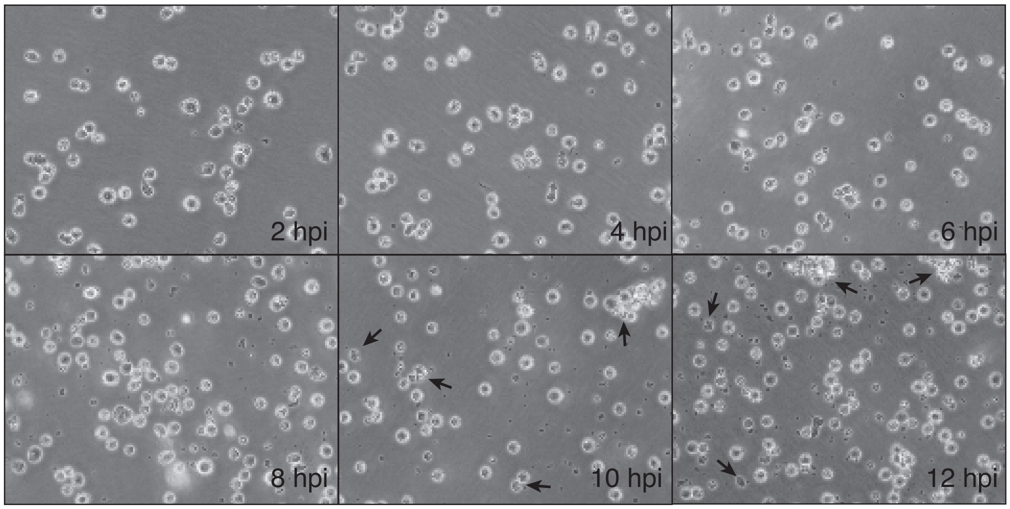

Table S1. Significantly enriched GO terms found in the exosome-like vesicle pellet of CrPV-infected S2 cells.

Name	GO ID	# Genes	Corrected p-value*
aerobic respiration	GO:0009060	13	2.24E-10
ATP biosynthetic process	GO:0006754	7	3.20E-03
ATP metabolic process	GO:0046034	29	4.98E-11
ATP synthesis coupled electron transport	GO:0042773	11	5.84E-11
ATP synthesis coupled proton transport	GO:0015986	7	2.99E-03
carbohydrate derivative metabolic process	GO:1901135	49	1.54E-06
carboxylic acid metabolic process	GO:0019752	52	3.39E-09
cellular lipid catabolic process	GO:0044242	6	0.03515077
cellular respiration	GO:0045333	25	5.17E-11
coenzyme metabolic process	GO:0006732	14	0.0336
cofactor metabolic process	GO:0051186	16	2.92E-03
electron transport chain	GO:0022900	15	6.40E-11
energy derivation by oxidation of organic compounds	GO:0015980	26	6.72E-11
fatty acid beta-oxidation	GO:0006635	5	0.02085517
fatty acid catabolic process	GO:0009062	6	0.03745574
fatty acid metabolic process	GO:0006631	8	0.0448
fatty acid oxidation	GO:0019395	6	0.03626667
generation of precursor metabolites and energy	GO:0006091	35	1.34E-09
hydrogen ion transmembrane transport	GO:1902600	16	0.01886316
hydrogen transport	GO:0006818	17	0.01775094
ion transport	GO:0006811	37	0.0192
lipid catabolic process	GO:0016042	6	0.03685161
lipid modification	GO:0030258	10	0.01710545
lipid oxidation	GO:0034440	6	0.0357
mitochondrial ATP synthesis coupled electron transport	GO:0042775	11	5.60E-11
mitochondrial electron transport, ubiquinol to cytochrome c	GO:0006122	6	3.28E-03
mitochondrial transport	GO:0006839	3	0.03755294
mitochondrion organization	GO:0007005	8	0.03811343
monocarboxylic acid catabolic process	GO:0072329	6	0.03461818
monocarboxylic acid metabolic process	GO:0032787	19	5.72E-03
monovalent inorganic cation transport	GO:0015672	19	0.02277966
nucleobase-containing small molecule metabolic process	GO:0055086	41	1.48E-08
nucleoside monophosphate biosynthetic process	GO:0009124	8	1.49E-10
nucleoside monophosphate metabolic process	GO:0009123	30	1.68E-10
nucleoside phosphate metabolic process	GO:0006753	38	2.69E-10
nucleoside triphosphate biosynthetic process	GO:0009142	8	1.03E-10

Table S1. Continued

nucleoside triphosphate metabolic process	GO:0009141	30	1.12E-10
nucleotide metabolic process	GO:0009117	38	1.92E-10
organic acid catabolic process	GO:0016054	9	0.01581176
organic acid metabolic process	GO:0006082	53	9.22E-09
organophosphate metabolic process	GO:0019637	45	1.27E-08
oxidation-reduction process	GO:0055114	60	4.80E-11
oxidative phosphorylation	GO:0006119	12	3.36E-10
phosphate-containing compound metabolic process	GO:0006796	73	5.36E-05
phosphorus metabolic process	GO:0006793	76	2.88E-06
phosphorylation	GO:0016310	42	7.02E-03
proton transport	GO:0015992	17	0.01742222
purine nucleoside monophosphate biosynthetic process	GO:0009127	8	1.22E-10
purine nucleoside triphosphate biosynthetic process	GO:0009145	8	8.96E-11
purine nucleoside triphosphate metabolic process	GO:0009144	30	9.60E-11
purine nucleotide biosynthetic process	GO:0006164	11	0.01097143
purine nucleotide metabolic process	GO:0006163	33	2.35E-08
purine ribonucleotide biosynthetic process	GO:0009152	10	3.13E-03
purine ribonucleotide metabolic process	GO:0009150	32	4.23E-09
purine-containing compound biosynthetic process	GO:0072522	11	0.010752
purine-containing compound metabolic process	GO:0072521	35	2.42E-07
respiratory electron transport chain	GO:0022904	13	6.11E-11
ribonucleoside monophosphate biosynthetic process	GO:0009156	8	8.40E-11
ribonucleoside monophosphate metabolic process	GO:0009161	30	7.91E-11
ribonucleoside triphosphate biosynthetic process	GO:0009201	8	7.07E-11
ribonucleoside triphosphate metabolic process	GO:0009199	30	7.47E-11
ribonucleotide biosynthetic process	GO:0009260	10	3.06E-03
ribonucleotide metabolic process	GO:0009259	32	4.10E-09
ribose phosphate metabolic process	GO:0019693	34	2.88E-08
small molecule catabolic process	GO:0044282	9	0.01550769
small molecule metabolic process	GO:0044281	86	5.38E-11
tricarboxylic acid cycle	GO:0006099	13	6.72E-10
tricarboxylic acid metabolic process	GO:0072350	13	4.48E-10

**Benjimini Hochberg-corrected enrichment p-value*

Table S2. CrPV peptides found in CrPV-infected versus mock S2 cell pellets

Peptide Sequence	Protein	PEP[a]	Score	Ratio[b] H/L
DTMSGETPEGK	CrPV ORF1	3.92E-05	111.61	6.9411
DVVFVDTLKDER	CrPV ORF1	4.49E-07	131.42	6.3489
FFMDHLDWFQQWK	CrPV ORF1	0.00042174	84.169	3.888
FLTYEEAIQGTGDDDFMK	CrPV ORF1	3.81E-58	304.7	32.6
GEEYNALDVTK	CrPV ORF1	8.19E-08	141.1	27.248
GEVGQLNGNLTR	CrPV ORF1	2.39E-08	153.1	0.66696
GTQGLYDEIHAWAK	CrPV ORF1	3.87E-06	119.54	6.5564
HYLDLDER	CrPV ORF1	0.0079989	71.379	24.383
IDNEIAVGTNVYSTDWER	CrPV ORF1	9.60E-14	173.36	7.0392
ITLDTETAAK	CrPV ORF1	0.0011923	85.731	8.955
IVIYDDAFQLK	CrPV ORF1	9.24E-08	138.98	12.927
LITTMLFPAK	CrPV ORF1	0.0017798	76.827	47.637
LNKDEAIDLSVYEFQK	CrPV ORF1	7.21E-05	96.666	26.698
MRFDDESATK	CrPV ORF1	0.0011234	96.229	48.381
MRPITVWLTGESGIGK	CrPV ORF1	0.033245	46.121	4.958
NTIDPNEILMMNINTAFR	CrPV ORF1	0.001242	73.927	5.795
QDWMGSGEQYDFTSQR	CrPV ORF1	2.93E-12	160.38	NaN
QVETEYWDGYNGQK	CrPV ORF1	2.97E-25	227.37	NaN
QVVLTQYNSMLDVNK	CrPV ORF1	1.51E-72	326.43	16.814
SLYEQVLLSPVK	CrPV ORF1	5.65E-18	199.09	1.642
TAVEVGSSGDSK	CrPV ORF1	0.00078656	85.355	5.7756
TFHQLQWLEAYASR	CrPV ORF1	6.37E-06	83.061	22.747
TKPNPEIFEVIR	CrPV ORF1	0.019918	48.524	3.427
VFSAGPQHFVVAFR	CrPV ORF1	2.38E-06	123.64	7.5992
WIDDGEPISYDEFAR	CrPV ORF1	3.69E-06	119.39	NaN
YGSTFGNGLLIVSPR	CrPV ORF1	1.01E-12	182.1	5.6464
ATFQDKQENSHIENEDK	CrPV ORF2	3.63E-26	229.96	58.739
DFLSRPIIIATNLWSSSDPVEK	CrPV ORF2	1.18E-08	102.45	8.1499
DSISATNHHITASFMR	CrPV ORF2	4.11E-13	179.17	11.162
EGLAGTSLDEMDLSR	CrPV ORF2	8.85E-73	336.79	99.182
EIVHFVSEGVTPSTTALPDIVNLSTNYLDMTTR	CrPV ORF2	5.08E-49	248.9	9.03
GSIVYTFK	CrPV ORF2	0.0001765	106.04	18.606
IATGDFTETEMR	CrPV ORF2	3.58E-08	150.81	260.6
IATGDFTETEMRK	CrPV ORF2	1.35E-08	152.4	86.686
ISFIPYYYNTTISTGTPDVSR	CrPV ORF2	4.03E-28	213.34	13.096
LFGFSKPTVQGK	CrPV ORF2	2.67E-05	106.01	49.839
LMLQYIPYAQYMPNR	CrPV ORF2	4.68E-12	203.4	10.801
MALSSTNEIETK	CrPV ORF2	4.05E-25	216.45	27.824
MANFDGMDMSHK	CrPV ORF2	7.77E-12	159.09	8.1089
MVAETQDGTGTPR	CrPV ORF2	2.54E-08	152.07	26.853
NEAQHGVHPISIDTHR	CrPV ORF2	1.81E-08	162.32	29.638
QENSHIENEDK	CrPV ORF2	0.047795	39.266	4.7674
QLYTANFPEVLISNAMYQDK	CrPV ORF2	2.74E-16	163.99	2.5511
RFGIFGDANTLQADGSSFVVAPFTVTSPTK	CrPV ORF2	1.48E-09	122.47	41.216
TNFTWFVR	CrPV ORF2	4.75E-07	136.75	92.426
TQEYSNNEDNR	CrPV ORF2	0.001169	80.239	NaN
TSDVTNTVLWDNYVSPFK	CrPV ORF2	1.10E-88	361.65	14.061
VKPYSATITDR	CrPV ORF2	1.22E-05	120.55	8.4676
VLSIPNYWDR	CrPV ORF2	2.86E-07	134.05	10.699
VQVNSQPFQQGR	CrPV ORF2	1.09E-07	136.63	14.45

[a]PEP = Posterior error probability

[b]Calculated peptide ratio based on heavy (H) labeled peptides from CrPV-infected S2 cells versus light (L) labeled peptides from mock-infected S2 cells.

Table S3. CrPV peptides found in ELVs from CrPV-infected versus mock S2 cells

Peptide Sequence	Protein	PEP[a]	Score	Ratio[b] H/L
ATFQDKQENSHIENEDK	CrPV ORF2	5.29E-165	362.51	1621.3
ATFQDKQENSHIENEDKR	CrPV ORF2	6.05E-166	369.97	1579.9
DSISATNHHITASFMR	CrPV ORF2	2.43E-08	102.29	83.8
EGLAGTSLDEMDLSR	CrPV ORF2	1.74E-82	293.94	NaN
GHSPPCLLTFSPR	CrPV ORF2	0.0074504	48.277	15.064
GSIVYTFK	CrPV ORF2	5.94E-06	136.16	4.2544
IATGDFTETEMR	CrPV ORF2	2.89E-36	226.2	454.46
IATGDFTETEMRK	CrPV ORF2	1.38E-47	240.1	769.1
IEAERTQEYSNNEDNR	CrPV ORF2	0.0012717	62.469	39.364
ISFIPYYYNTTISTGTPDVSR	CrPV ORF2	6.82E-06	112.03	NaN
ISNNWSPQAMCIGEK	CrPV ORF2	0.020345	54.199	1.4312
KIEAERTQEYSNNEDNR	CrPV ORF2	4.22E-14	129.88	149.41
MALSSTNEIETK	CrPV ORF2	6.30E-13	172.8	30.2
MANFDGMDMSHK	CrPV ORF2	0.00050574	87.184	75.584
MVAETQDGTGTPR	CrPV ORF2	1.09E-09	150.61	13.988
NEAQHGVHPISIDTHR	CrPV ORF2	1.85E-08	106.34	230.81
QLYTANFPEVLISNAMYQDK	CrPV ORF2	1.93E-93	275.83	NaN
TSDVTNTVLWDNYVSPFK	CrPV ORF2	5.32E-122	315.73	6.1475
VKPYSATITDR	CrPV ORF2	0.00029335	114.24	8.2293
VKPYSATITDRFR	CrPV ORF2	5.00E-07	123.95	221.54
VQVNSQPFQQGR	CrPV ORF2	2.83E-36	226.39	10.093

[a]PEP = Posterior error probability

[b]Calculated peptide ratio based on heavy (H) labeled peptides from CrPV-infected S2 cells versus light (L) labeled peptides from mock-infected S2 cells.
